# Special Diets in Infants and Children and Impact on Gut Microbioma

**DOI:** 10.3390/nu14153198

**Published:** 2022-08-04

**Authors:** Elisabetta Di Profio, Vittoria Carlotta Magenes, Giulia Fiore, Marta Agostinelli, Alice La Mendola, Miriam Acunzo, Ruggiero Francavilla, Flavia Indrio, Alessandra Bosetti, Enza D’Auria, Elisa Borghi, Gianvincenzo Zuccotti, Elvira Verduci

**Affiliations:** 1 Department of Pediatrics, Vittore Buzzi Children’s Hospital, Università di Milano, 20154 Milan, Italy; 2 Pediatric Section, Department of Interdisciplinary Medicine, University of Bari “Aldo Moro”, 70121 Bari, Italy; 3 Department of Medical and Surgical Sciences, University of Foggia, 71122 Foggia, Italy; 4 Department of Health Sciences, University of Milan, 20142 Milan, Italy; 5 Department of Biomedical and Clinical Sciences L. Sacco, University of Milan, 20144 Milan, Italy; 6 Pediatric Clinical Research Center, Fondazione Romeo ed Enrica Invernizzi, University of Milan, 20122 Milan, Italy

**Keywords:** gut microbiota, infants, children, standard diets, special diets, dietary pattern

## Abstract

Gut microbiota is a complex system that starts to take shape early in life. Several factors influence the rise of microbial gut colonization, such as term and mode of delivery, exposure to antibiotics, maternal diet, presence of siblings and family members, pets, genetics, local environment, and geographical location. Breastfeeding, complementary feeding, and later dietary patterns during infancy and toddlerhood are major players in the proper development of microbial communities. Nonetheless, if dysbiosis occurs, gut microbiota may remain impaired throughout life, leading to deleterious consequences, such as greater predisposition to non-communicable diseases, more susceptible immune system and altered gut–brain axis. Children with specific diseases (i.e., food allergies, inborn errors of metabolism, celiac disease) need a special formula and later a special diet, excluding certain foods or nutrients. We searched on PubMed/Medline, Scopus and Embase for relevant pediatric studies published over the last twenty years on gut microbiota dietary patterns and excluded case reports or series and letters. The aim of this review is to highlight the changes in the gut microbiota in infants and children fed with special formula or diets for therapeutic requirements and, its potential health implications, with respect to gut microbiota under standard diets.

## 1. Gut Microbiota in Infants and Children: What Influences It?

Gut microbiota is a complex community of microorganisms that live in the gastrointestinal (GI) tract of humans and animals [1,2]. Gut microbiota consists of more than 1500 species, distributed in more than 50 different phyla [3]. The most abundant phyla were reported to be Bacteroidetes and Firmicutes, followed by Proteobacteria, Fusobacteria, Tenericutes, Actinobacteria, and Verrucomicrobia, which make up to 90% of the total microbial population in humans [2,4]. Concerning diversity, the developing infant microbiota progressively undergoes an increase in within-individual diversity (the so-called alpha diversity) and a decrease in between-individual diversity (beta diversity) [5,6,7]. Several factors influence the rise of microbial gut colonization, such as term and mode of birth, exposure to antibiotics, presence of pets, siblings and family members, genetics, local environment, geographical location, and maternal and infant/toddler diet, with diet being a major player [5,8,9]. Aagaard et al. [10] have shown that contrary to what was previously thought, the placenta itself already contains microbes, similar to those in the oral cavity). Nevertheless, the pivotal colonization event occurs at birth. The newborn external body surfaces and GI tract are then rapidly colonized by several microorganisms, reaching up to 10^8^ microorganisms per gram of feces in hours to days [11]. Over weeks to months, this number increases, reaching 10^11^–10^12^ [5,12], values that are comparable to the adult microbial densities [13].

As the infant gets older, their exposure to new microbes increases, but the influence of these exposures to the microbial gut composition decreases [5]. Extensive research has focused on dietetic influence on microbiota, specifically on breast milk versus formulas, whereas much less is known about the effects of complementary feeding on microbiota composition [14]. Around 4–6 months of age, infants pass from exclusive milk-feeding to eating family foods, usually referred to as the complementary feeding (CF) period [15].

Dietary fibers and incompletely digested proteins are the main sources of energy for microbes that reside in the gut, and tare he most involved in shaping the microbial composition [14]. The main end products of dietary fiber fermentation are the short chain fatty acids (SCFAs) (i.e., acetate, propionate, and butyrate) [16]. SCFAs influence intestinal barrier function, metabolism, immune and nervous systems [8,17].

Considering the great impact of the diet on gut microbiota, a deep knowledge of the potential microbe-mediated host effects of feeding mode in infancy and childhood is required [8]. Moreover, specific dietary interventions utilized in children should also be evaluated in relation to their potential effects on the gut microbiome. The aim of this review is to highlight changes in the gut microbiota in infants and children on a special diet for therapeutic requirements and potential health implications.

## 2. Methods

We performed a narrative review according to the literature in English over the past 20 years. The authors independently identified the most relevant published studies, including original papers, metanalysis, clinical trials, and reviews. Case reports or series and letters were excluded. Pediatric literature was considered and, regarding pathogenetic mechanisms, animal studies were also included. Papers published up to June 2022 were selected by means of the following keywords (alone or in combination): infants, toddlers, childhood, gut microbiota, dietary pattern, breast milk, formula milk, extensively hydrolyzed formulas, amino acid formulas, cow’s milk protein allergy, complementary feeding, Mediterranean diet, Nordic diet, Atlantic diet, Japanese diet, Vegetarian diet, avoidance diet, food allergy diet, phenylketonuria diet, ketogenic diet, inborn errors of metabolism diet, gluten-free diet, low-fodmap diet. The following electronic databases were used: PubMed/Medline, Scopus, and EMBASE.

## 3. Gut Microbiota in Infants and Toddlers under Standard Diets

### 3.1. Breast Milk vs. Formula Milk and Gut Microbiota

Breast milk, composed of lactose, lipids, proteins, and more than 200 different human milk oligosaccharides (HMOs) [18], is recommended as the first nutrition during infancy, providing all the necessary nutrients to support growth and development [19,20,21]. HMOs are not hydrolyzed by gastrointestinal saccharolytic enzymes; thus, they reach the colon intact, where they serve as metabolic substrates for gut bifidobacteria [8]. Some bifidobacterial species (e.g., *B. bifidum*, *B. breve* and subsp. *infantis*) possess specific membrane transporters and saccharolytic enzymes that are capable of cleaving and internalizing HMOs, producing lactate, acetate, formate, and 1,2-propanediol [22]. The end products of HMOs’ metabolism have potential beneficial host-health effects. Indeed, acetate and lactate have been reported to suppress the growth of opportunistic pathogens belonging to *Clostridiaceae*, *Enterobacteriaceae*, and *Staphylococcaceae* [8,23]. The lack of *Bifidobacterium* spp. has been instead associated with immune dysregulation [24], asthma [25], and autoimmune diseases [26]. Interestingly, Alcon-Giner et al. conducted a longitudinal study to evaluate the gut microbiota composition in two groups of preterm infants (101 orally supplemented with *Bifidobacterium* and *Lactobacillus* -Bif/Lacto- species and 133 non-supplemented). They observed enrichment in *Bifidobacterium* and a lower abundance of opportunistic pathogens in the Bif/Lacto group [27]. Moreover, fecal acetate positively correlated with *Bifidobacterium* relative abundance [27].

Overall, HMO and lactose promote the GI microbiota development and stimulated the infant immune system through growth factors, cytokines and immunoglobulins, with a positive impact on growth, neurodevelopment and metabolism [28].

Exclusively formula-fed (FF) infants possess different microbiota, characterized by a lower abundance of HMO-utilizing *Bifidobacterium* species and increased *Clostridium* species (e.g., *C. difficile* and *C. perfringens*) and *Enterobacteriaceae* (e.g., *Escherichia coli*) [29,30]. These observations seem to correlate with the lack of HMOs and the higher protein content in milk formulas [8]. Recently, Berger et al., in a double-blinded randomized controlled trial, evaluated whether supplementation of infant formula with HMOs, (specifically 2′-fucosyl-lactose-2′FL- and lacto-N-neotetraose-LNnT) could influence the gut microbiota [31]. Notably, recent studies have evaluated the effect of infant formula supplemented with synthetic HMOs (e.g., 2′FL and LNnT) in order to mimic a BF-like gut metabolism and microbial colonization [32]. Moreover, the researchers compared HMO-supplemented and un-supplemented formula groups with BF infants [31,33]. The intervention group led to a microbiota pattern that was more similar to that of the breast-fed (BF) infants, with higher levels of *Bifidobacterium* spp. and a lower relative abundance of *Clostridiaceae* and *Enterobacteriaceae,* compared with infants fed with un-supplemented formula [31].

Many infant formulas are, nowadays, supplemented with fructo-oligo-saccharides (FOS) and/or galacto-oligosaccharides (GOS), although these are not selected as maternal HMOs as they can be utilized by most *Bifidobacterium* species (e.g., *B. adolescentis* and *B. catenulatum*, typical of adulthood) [29,30]. Studies comparing the fecal metabolome in exclusively BF and exclusively FF infants (even when the formula contains galacto-oligosaccharides) show that proteolytic rather than saccharolytic metabolism dominates in the FF gut [34,35]. Indeed, in BF infants, remnants of HMO metabolism are mainly present, while in FF infants, the main observed products are derived from protein fermentation [34,35]. Thus, the authors evaluated the intestinal fermentation capability, which might be influenced by early diet [34]. The different fecal microbiota were also related to the differences in the fecal water % and in the concentration of microbial by-products in the stool [34]. In FF infants, limited oligosaccharides and high protein levels were present [34]. The difference in terms of fecal microbial taxonomic profiles of the milk fat globule membrane (MFGM) supplemented with experimental formula (EF) and standard formula (SF) was moderate, with respect to the difference between BF and FF infants [34]. However, to confirm these results, further studies are needed.

**Figure 1:**
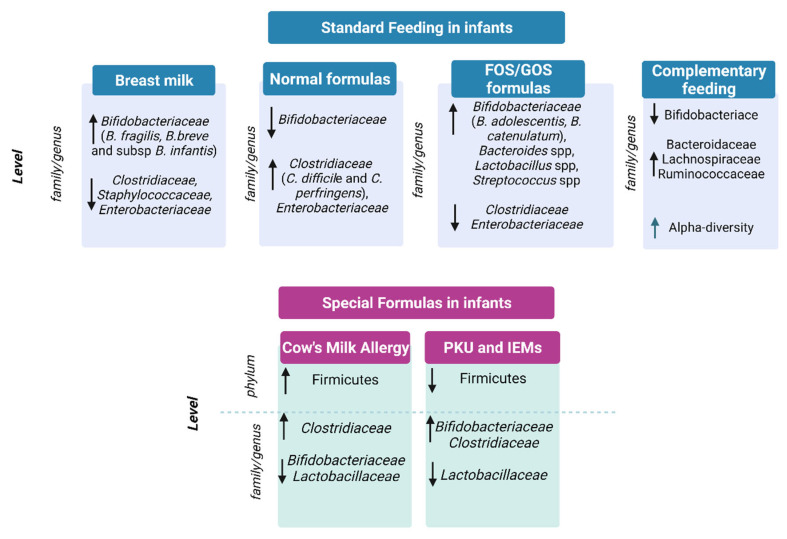
Gut microbiota in standard feeding vs. special formula-fed infants. ↓= decrease in family/genus or phylum; ↑ = increase in genus/family or phylum.

In conclusion, breastfeeding, mainly due to the presence of HMOs, promotes the colonization of beneficial bifidobacteria and is able to support immune system development and to prevent infections, as depicted in Figure 1 [8,23,36,37,38]. On the contrary, milk formulas have been correlated to the growth of opportunistic pathogens and to proteolytic gut metabolism, potentially causing adverse health effects [39,40,41,42].

### 3.2. Gut Microbiota and Complementary Feeding

Complementary feeding is a period characterized by a shift from an exclusive milk diet to a mixed diet, including food consumption and it goes along with a phase of remarkable changes in the gut microbiota, of which the most relevant is the rapid decline in HMO-degrading *Bifidobacterium* species [43]. Moreover, alpha diversity increases and higher levels of *Bacteroidaceae*, *Lachnospiraceae*, and *Ruminococcaceae* species are present. This mirrors the higher diversity and complexity of the diet, due to the introduction of fibers (from fruits, vegetables and cereals) and new protein sources (as meats, dairy products, and legumes) [8,14].

Coherently, Differding et al. associated *Bilophila wadsworthia* and *Roseburia* spp. with early complementary feeding practice (e.g., consumption of solids or non-water/formula liquids at or before 3 months) [44]. Leong et al., in a randomized controlled trial, compared the gut microbiota composition of children weaned with a “baby-led” approach of complementary feeding with traditional spoon-fed infants [45]. The baby-led weaning approach was associated with lower alpha diversity and lower levels of *Lachnospiraceae* (*Roseburia facies* and *Eubacterium rectale*) and *Ruminococcaceae* (*Faecalibacterium prausnitzii*) [45]. The multinational European INFABIO study (*n* = 605 infants) found that early introduction of complementary foods was associated with a decrease in bifidobacteria (sp. unknown) [46]. Specifically, the study confirmed that the initial feeding method influenced the relative abundance of the *Clostridium leptum* group, *Clostridioides difficile* and *C perfringens*, and *bifidobacteria* dominated the feces of initially breast-fed infants [46].

Some complementary foods, such as rye bread, cheese, and meat products, have been specifically correlated to this increase in diversification of the infant gut microbiota [47]. Importantly, complementary feeding progression has also been correlated to higher levels of several *Lachnospiraceae* and *Ruminococcaceae*, and to a lower abundance in *Bifidobacterium* spp. [14]. This underlines the transition from a bifidobacteria-rich gut community toward a more diverse gut microbial community, rich in butyrate-, propionate-, and BCFA-producing bacteria [14].

Concerning the different food introduced, meat causes significantly increased levels of genera that belong to *Lachnospiraceae*, which are potential short-chain fatty acid producers with respect to dairy [48]. Recently, a comparison between refined grain cereal products and whole grain cereal products as the first complementary food showed a significant increase in *Bacteroides* and *Lachnoclostridium (Lachnospiraceae* family) and a decrease in *Escherichia* (*Enterobacteriaceae* family) in the whole grain cereal group [47].

The results from these studies are encouraging, but in order to define a direct causal relationship between certain complementary foods and specific microbial changes, further evidence is needed.

## 4. Gut Microbiota in Infants under Special Diets

### 4.1. Special Formulas for the Treatment of Cow’s Milk Allergy

Cow’s milk protein allergy (CMA) is the most common food allergy in the pediatric population [49]. The use of extensively hydrolyzed formulas (eHF) is recommended as the first choice for the treatment of infants and children with confirmed CMA [50]. Amino acid-based formulas (AAFs) are also available and are considered the safest dietary strategy for infants with severe CMA [51,52]. Hydrolyzed formulas minimize antigen contact compared to whole-protein formulas, but due to the presence of sparse small peptide fragments, it also may favor immune tolerance by different mechanisms, acting on barrier reinforcement, neuro-immune pathways, and on the resident microbial community [53]. Indeed, the peptides derived from protein hydrolysates can act locally in the gut, modulating the microbiota. Dysbiosis induces functional alterations that result in aberrant Th2 responses toward an allergic, rather than a tolerogenic, response [54]. Conversely, recent data suggest that the use of AAF, the only totally non-allergenic formulas, exclusively providing nitrogen equivalent proteins as free amino acids, may delay the acquisition of immune tolerance [55], since they are completely devoid of allergenic antigenic epitopes [50,56]. Paparo et al. showed the absence of AAF-related effects on the intestinal barrier, Th1/Th2 cytokine response, and regulatory T cells (Treg) activation in vitro [57]. The onset of oral tolerance occurs during the critical early stages of immune development and it depends not only on genetic factors, but also on environmental factors, including appropriate intestinal bacterial colonization [58]. Research on the development of oral tolerance has recently focused on the influence of host–microbe interactions on immune function, in particular on the ability of specific gut microbes to promote tolerance to food antigens [59,60,61]. In this respect, it has been reported that supplementation with *Lactobacilllus rhamnosus* GG (in an extensively hydrolyzed casein formula, eHCF) in infants with CMA stimulates immune tolerance [55,62,63], and some eHFs were supplemented with specific probiotic strains, which in turn may act on immunomodulation, in addition to the small peptides. Berni Canani et al. found that dietary management with an eHCF formula supplemented with the probiotic *Lactobacillus rhamnosus* GG (LGG) results in a higher rate of tolerance acquisition in children with IgE-mediated CMA, compared with non-supplemented eHCF or non-casein-based formulas [55]. In addition, in a well-characterized population of children with IgE-mediated CMA, it was shown that eHCF + LGG is superior to eHCF alone in preventing other allergic manifestations over a 36-month period [64]. The gut microbiota produces metabolites, such as SCFAs, beneficial for the colonic environment, which participate in improving barrier function, increasing Treg cells, and protecting against pathogenic bacteria colonization [65,66]. Another of the hypothesized mechanisms for an increased immune tolerance is the enrichment of butyrate-producing bacteria, as butyrate stimulates the development of Treg. Treg cells play a pivotal role in the development of oral tolerance [67,68,69] and their reduction has been associated with an allergic phenotype [70,71]. Ruohtula and colleagues found that an increase in highly activated Treg cells is associated with colonization of butyrate-producing bacilli and suggested a narrow window of opportunity (from birth to 3 months of age) for primary prevention of atopic diseases [72]. eHFs favour butyrate production, as highlighted by Car RennKok et al., who compared healthy term breastfed infants vs. infants fed with eHF or AAF. The authors found significant differences between the groups in beta-diversity at day 60, and a similar gut microbiota composition in the formula groups compared to the breastfed group. The relative abundance of *Bifidobacterium* spp. increased over time and was significantly enriched at day 60 in the human milk group. In contrast, a significant increase in Firmicutes members was found at day 60 in the formula groups, as well as butyrate-producing species in the eHF group. Butyrate increased significantly from baseline to day 60 in the eHF group and it was significantly higher in the study formula groups than in the human milk at day 60 [73]. Berni Canani et al. performed gut microbiota and oligotyping analysis on stool samples collected from healthy infants and infants with CMA before and after treatment with eHCF with or without LGG supplementation. They found that the infants who became tolerant early showed a significant increase in fecal butyrate levels and that eHCF + LGG promotes tolerance in infants with CMA, in part, by influencing the structure of the gut bacterial community at the strain level [63]. The same authors showed that children with non-IgE-mediated CMA had significantly lower fecal butyrate concentrations than healthy controls at the baseline, and dietary regimes (eHCF or eHCF + LGG) were associated with a significant increase in butyrate concentrations, especially in children treated with eHCF + LGG. In particular, the treatment with eHCF + LGG appears to restore the composition and structure of the subgenus *Bacteroides*, which shows similar diversity to that of healthy controls. Treatment with eHCF + LGG significantly increased butyrate production, and this change correlated with the enrichment of potential SCFA producers and selected *Bacteroides* oligotypes [74].

Besides probiotics, hydrolyzed formulas were also supplemented with oligosaccharides [75]. The effects of a formula supplemented with 2 HMOs-2′fucosyllactose (2′FL) and 0.5 g/L lacto-N-neotetraose (LNnT) on infant growth, tolerance and morbidity have been evaluated vs. non-supplemented formula milk [75]. The authors observed a similar weight gain in the two groups at 4 months, and similar digestive symptoms and behavioral patterns between the groups [76]. The safety and hypoallergenicity of an eHF supplemented with 2′FL and LNnT compared with a standard eHF were successfully assessed by Nowak-Wegrzyn and coworkers [77].

The CINNAMON study, published in 2022, confirmed the tolerance and safety data of a formula hydrolyzed with 2′FL and LNnT in a pediatric population with CMA [78]. While no differences were found in the anthropometric parameters compared to the fortified formula, a lower α-diversity was observed in the intestinal microbiota of the children receiving the supplemented formula [78]. Further studies are needed to fully elucidate the effects of formula milk supplemented with oligosaccharides.

Regarding synbiotics, in a randomized study, either an AAF or an AAF with synbiotics (oligofructose, long-chain inulin, acid oligosaccharides and *Bifidobacterium breve* M-16V) were administered to 110 children diagnosed with CMA. No differences were found in the growth pattern and allergic symptoms between the two groups, but synbiotics increased bifidobacteria and reduced *C. hystoliticum*, *Eubacterium rectale* and *Clostridium coccoides* clostridial groups’ relative abundances in the fecal samples [79]. A similar study design was conducted by Candy et al. in 2017 in a group of children with non-IgE-mediated CMA by comparing their fecal samples to healthy BF infants. The test formula was an AAF supplemented with a prebiotic mixture of fructo-oligosaccharides and the probiotic strain *Bifidobacterium breve* M-16V. The authors found that the addition of specific synbiotics leads to levels of bifidobacteria, *E. rectale* and *C. coccoides* group similar to those of BF infants, thus improving the fecal microbiota of infants with suspected non-IgE-mediated CMA [80].

The possible influence of eHF formulas in promoting butyrate-producing species, and consequently butyrate production itself, is of great interest in the development of oral tolerance in infants with CMA. In addition, the role of probiotic-supplemented formulas, particularly LGGs, in the acquisition of immune tolerance is emerging. Future studies are required to elucidate the role of both synbiotics and HMOs in the pediatric population with CMA.

### 4.2. Special Formula for Functional Gastrointestinal Symptoms

Gastrointestinal symptoms, including frequent regurgitation, vomiting, and colicky, are very common in infants, and represent the most frequent cause of formula change in the first six months of age.

These symptoms can overlap non-IgE CMA ones, making it difficult to distinguish between them in clinical practice. However, it is very important to differentiate between these conditions as CMA is confirmed by oral food challenge only in a small percentage of infants [81] and it requires the use of an eHF, according to the guidelines [50]. A clinical score known as CoMiSS (cow’s milk related symptoms score) has been developed as a clinical tool aimed at increasing the awareness of health care professionals in identifying clinical manifestations possibly related to cow’s milk (CM) intake [82]. In a study comparing the effects of two eHF supplemented with probiotics in infants with suspected CMA, an improvement in all the items was observed after dietary intervention [83]. The observed effects on intestinal microbes were, as expected, an increase in bifidobacteria in the group with eHF supplemented with bifidobacteria, and enrichment in lactobacilli in the group with eHF supplemented with lactobacilli. Both formulas were equally effective and safe.

The effective response to hydrolyzed formulas in both functional gastrointestinal disorders and non-IgE CMA may be due to immunological mechanisms, as well as to improved gastric emptying [54,84]. Furthermore, a CM-free diet reduces mast cell infiltration and normalizes immune–nerve interactions and motor function [54].

Comparing a thickened eHF vs. a standard eHF on 77 infants with suspected CMA and bothersome regurgitation or vomiting (more than five episodes per day), a reduction in regurgitation was observed, both upon eHF use (after one month of intervention) and, to a greater extent, upon thickened formula intake [85].

In a French group of 30 infants with proven CMA by a double-blind dietary test (70% with positive IgE tests), the administration of a casein-based thickened eHF significantly decreased both regurgitation and clinical crying scores after only 14 days [86].

In 2014, Vandenplas et al. analyzed the effect of two casein-based eHFs in 72 formula-fed infants with symptoms suspected to be related to CMA and found an overall clinical improvement after one month, especially in infants with a positive CM challenge (65%) [87].

In 2018, a Cochrane review that focused on the role of diet modifications in infants with colic was published. The review, which collected 15 randomized controlled trials for a total of 1121 infants, concluded that 25% of infants with moderate to severe colicky symptoms experienced a significant reduction in the crying time on a CM-free diet compared to the group with intact CM protein intake [88].

Literature data, although limited, suggest that the use of a partially hydrolyzed infant formula may be of benefit in reducing infant colic in formula-fed infants [89]. The use of partially hydrolyzed, lactose-reduced whey protein formulas with prebiotic oligosaccharides and probiotics in infants who are not suspected of suffering from CMA is recommended by some authors [90]. Moreover, also in non-breast-fed infants without a CMA, a formula based on partially hydrolyzed, lactose-reduced whey protein with prebiotic oligosaccharides and/or probiotics may be considered, case by case [90].

Xinias et al. showed the efficacy in the management of infant colic of a partial hydrolysate of whey with reduced lactose, *Bifidobacterium lactis* BB12, and galacto-oligosaccharides [91]. In this regard, colicky children show less diversity and stability in the microbiome and gastrointestinal colonization develops more slowly [92]. The gutmicrobiota of these children has low levels of bifidobacteria and lactobacilli and a reduced number of butyrate-producing bacterial species [93,94].

Two systematic reviews of small randomized trials suggest that eHF formulas may reduce distress in infants with colic [95,96]. However, methodological biases hamper drawing recommendations regarding the use of hydrolyzed formulas to treat this condition. Furthermore, Brink et al. evaluated the fecal microbiota at 3, 6 and 12 months in BF infants compared to those FF with whole-food protein formula and those FF with soy-based formula. They found, in all the considered time points, a greater α-diversity in the soy formula (SF) fed infants and a 2.6- to 5-fold reduction in *Bifidobacterium* spp. levels in SF group, compared to BF infants up to 1 year of age [97]. These results are in agreement with a previous study on a cohort of infants up to 8 months of age, reporting a higher α-diversity in the SF-fed subjects [98]. In addition, a previously unidentified genus of *Ruminococcaceae* was enriched in the SF group compared to milk-based formula or BF groups at 3 months of age, and unidentified genera of the order Clostridiales were more numerous in the SF group than the other two at 6 and 9 months of age [97], confirming the data previously observed in the animal model [39]. Gastro-intestinal disorders may cause alterations in gut microbial diversity. On the other hand, the use of formulas to treat these pathologies is suggested to impact the neonatal microbiota. For example, soy-based formulas seem to increase alpha-diversity at the expense of a reduction in bifidobacteria. Current studies are inconclusive, and a deeper comprehension is needed to draw guidelines for the management of GI disorders.

### 4.3. Phenylketonuria and Inborn Errors of Metabolism Formulas

Studies on the microbiome and its influence in patients with inborn errors of metabolism formulas (IEMs) mostly focused on phenylketonuria (PKU). To date, only one open-label, single-arm intervention pilot study in 2011 has evaluated the tolerance and efficacy of adding a prebiotic oligosaccharide mixture (scGOS/lcFOS) to a protein replacement suitable for children with PKU in 9 infants with PKU diagnosis. At the end of the observation period (8-weeks), all children had microbiota dominated by bifidobacteria, unchanged from the baseline. No child showed abnormally high levels of *Clostridium histolyticum*/*lituseburense* or potentially pathogenic *Enterobacteriaceae* at any time during the study. A significant reduction in median stool pH from the baseline was observed at week 4 (pH from 6.79 to 5.83), but this observation was not confirmed at week 8 [99]. To date, there are no other clinical studies in pediatric populations with IEMs on the influence of special formulas on the gut microbiota.

## 5. Gut Microbiota in Childhood

### Standard Diets during Childhood

#### Mediterranean Diet, Japanese Diet, Nordic Diet and Atlantic Diet

During the pediatric age, the eating environment is crucial for acquiring correct eating habits that might have a positive effect on the gut microbiota and on host health. It is universally recognized that certain dietary patterns are protective against non-communicable diseases (NCDs). Healthy children, who do not need special restrictions, acquire the eating habits of the environment in which they grow up.

The Mediterranean diet (MD), characterized by a low presence of processed foods and a high intake of products rich in fibers or antioxidants, was the first to be studied for its preventive characteristics against NCD development [100]. MD adherence varies widely within the Mediterranean countries and during pediatric ages [101,102].

**Figure 2:**
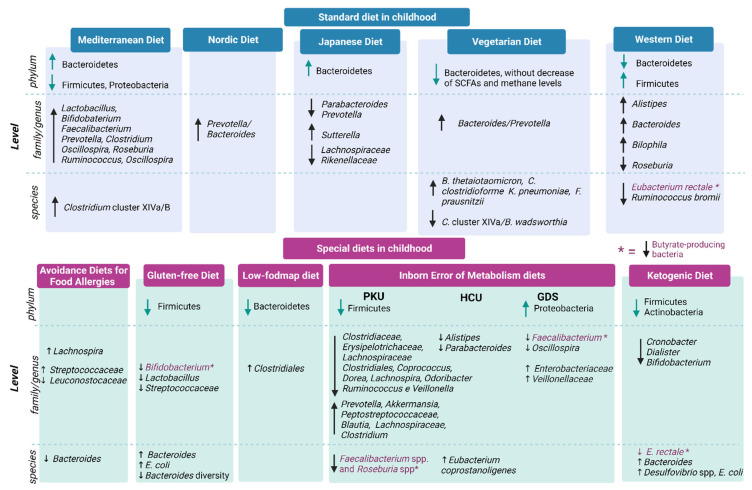
Gut microbiota composition in children with standard diets vs. special diets. ↓= decrease in family/genus or phylum; ↑ = increase in genus/family or phylum.

Robust literature data demonstrate that the MD can lead to higher gut microbiota diversity [100], which means an increase in Bacteroidetes, lactobacilli, bifidobacteria, *Faecalibacterium* spp., *Clostridium* cluster *XIVa* and a decrease in Firmicutes and Proteobacteria [103] (see Figure 2).

A further positive dietary pattern, commonly adopted in Nordic countries, is the so-called Nordic diet (ND) [104]. ND consumers have a high intake of fiber and their microbial community correlates with high SCFA (butyrate, acetate, propionate) production, with positive effects on cardiometabolic outcomes. In addition, they eat a large number of phenolic compounds (particularly hippuric acid) that are positively associated with *F. prausnitzii,* known for its ability to produce butyrate. Moreover, other compounds, such as furan fatty acids, trimethylated compounds, and indole-metabolites, although not yet fully investigated, have been linked to ND and gut microbiota function. [105].

The traditional Japanese diet (JD), also called “Washoku”, has been raised to have beneficial properties for the microbiota. Indeed, JD is rich in minimally processed, fresh, seasonal foods and fermented food (shoyu, koji, natto, tsukemono, fermented seafood), which are centrepieces of JD [106].

This dietary pattern seems to promote a positive balance between Firmicutes and Bacteriodetes and improve both liver function and the immune system [107].

In the last few years, the Southern European Atlantic diet (SEAD) has been recognized as a traditional dietary pattern [108]. This diet belongs to the Atlantic region [109] and it has been recently linked to epigenetic regulation also involving the gut microbiota [110].

Several studies [111,112,113,114] have also showed modulation of the gut microbiota also by the vegetarian diet (VD) (see Figure 2). However, there are still no comprehensive conclusions due to several confounding factors. For instance, SCFA production and methane levels were found unchanged, despite lower counts of *Bifidobacterium* and *Bacteroides* species. Indeed, some microbiome-modulating molecules, such as polyphenols, are found in high amounts in plant-based diets and should be taken into account to clarify the complex mechanisms and interrelationships between vegan/vegetarian diets and gut microbiota.

A high degree of cooperation and mutualistic behaviors occur between different bacteria during the fermentation of dietary fibers [115]. At the same time, diverse bacterial populations use different fermentation by-products as their main energy source. Both the composition and the metabolic activity of gut microbes, largely driven by fiber fermentation, are important players in the development of chronic NCDs and in infection incidence and severity [116].

Although research has shown the importance of such eating behaviors, globally, even in rural areas of developing countries, there is an increasing trend in the intake of certain foods typical of the so-called Western diet (WD) [117].

The WD is characterized by a high presence of sugar-rich, highly refined foods, rich in saturated fats, and animal proteins and a low in vegetables, plant proteins, fish and foods rich in antioxidants [118]. WD is associated with negative outcomes for NCDs and to obesity development [119]. Animal models confirm this observation. For example, in mice fed with WD since early life, long-lasting neurocognitive impairments were found [120].

The importance of standard diets (MD, JD, ND, and VD) has been settled not only for healthy growth but also in relation to the establishment of a beneficial gut microbiota. A dietary regimen excluding specific nutrients or foods for life can be necessary when some diseases occur, such as in the presence of coeliac disease, allergies or metabolic inborn errors (IEMs). In the following paragraphs, we will explain whether these ‘diet deprivations’ can affect the composition of the child’s microbiota and its possible implications.

## 6. Main Special Diets during Childhood

### 6.1. Avoidance Diets for Food Allergies

A food allergy (FA) is a hypersensitivity reaction to a specific food antigen and is classified as immunoglobulin E (IgE)-mediated and non-IgE-mediated [121]. Resident microbiota may influence the generation of food antigen-specific regulatory T (Treg) cells. [59,122]. In patients with FA, induction of Treg cells is believed to be compromised and replaced by the generation of unique antigen-specific Th2 cells that drive IgE class-switching and expansion of allergic effector cells [123]. Despite ongoing investigations, the immunopathogenic mechanisms underlying non-IgE-mediated food allergies are still largely unknown [124]. Very common food allergens for children are milk and eggs. In fact, the management of FA includes allergen avoidance and emergency treatment [125]. For children with a severe IgE-mediated food allergy to cow’s milk, egg and peanut, oral immunotherapy can be considered, although side effects limit the routine application of this treatment [126].

In the past few years, the crucial role of gut microbiota in host immunity has become clear. Changes in the quantity or quality of gut microbes, a condition known as dysbiosis, can indeed affect tolerance development and raise susceptibility to food allergies [127,128]. Goldberg et al. demonstrated the presence of a “microbial signature” in subjects with persistent FA [129] in a study that recruited 233 patients with different FA vs. healthy controls. The gut microbiota composition of the allergic patients was significantly different compared to the age-matched controls both in terms of α-diversity and β-diversity. *Prevotella copri*, for example, seemed to be more abundant in the healthy group [129]. The cause for changes in the *P. copri* relative abundance in the different FAs is not fully understood. The authors also compared fecal SCFA concentrations. Acetate was significantly lower in the allergic patients compared to the control group, which might result from the lack of microbial species, such as *P*. *copri*, which synthesize acetate and propionate as metabolites from complex carbohydrate fermentation [129]. This study also demonstrates that patients with different FA had similarities in their microbiota composition, strengthening the association of the mere presence of FA with a microbial signature, rather than different diets.

Regarding food allergies, CMA is the most common FA in the first years of life. Strategies for the management of CMA include avoidance of cow’s milk protein from a child’s diet [130]. The immune mechanism of CMA can be IgE-mediated or non-IgE-mediated (cell mediated). A total of 20% of children with CMA acquire tolerance in the first year of life, 50% by 5 years, and 75% by early adolescence [130,131]. However, even though there is an overlapping feature in IgE and non-IgE-mediated CMA children, compared to healthy children, a significant enrichment in *Bacteroides* was observed in non-IgE-mediated and to a lesser extent in IgE-mediated CMA microbial profiles. *Bacteroides* species are reported to alter gut permeability [132] and their increase has been associated with peanut and tree nut allergy and other atopic manifestations [132,133].

Byniavanic et al. investigated the role of microbiota composition on tolerance achievement in children with CMA. They longitudinally followed 226 children with CMA from the age of 3–16 months to children aged 8 years old [134]. The authors found an enrichment of Firmicutes and in particular of clostridia in the 3–6-month-old gut microbiome of subjects whose milk allergy resolved by age 8 years. Thus, the authors suggested that clostridia could be studied as probiotic candidates for milk allergy therapy.

A recent metanalysis also affirms that probiotics were associated with a higher rate of acquisition of tolerance to cow’s milk protein at the end of 3 years compared with the placebo [135]. However, more high-quality studies with long-term follow-ups are needed to assess the potential of probiotics as an intervention for gut microbiota of children with CMA.

Egg allergy affects 0.5% to 2.5% of young children [136]. To date, only one study aimed at comparing the gut microbiota composition in children with an egg allergy with age-matched controls has been carried out [137]. This study found an increased α-diversity and relative abundance of *Lachnospiraceae*, *Streptococcaceae* families in children with an egg allergy, while *Leuconostocaceae* were enriched in the controls. Similarly, in children with an egg allergy, increased gut bacterial diversity was associated with egg sensitization and with enrichment of genera from *Lachnospiraceae* and *Ruminococcaceae* in egg sensitized children. However, in this population, they did not find associations between early-life gut microbiota and resolution of egg allergy by age 8 years [137].

Overall, FA patients show reduced richness and evenness in their gut microbiota. Moreover, they have a reduction in SCFA butyrate-producing bacteria, and no core gut microbiota composition was reported among different FA populations.

### 6.2. Gluten-Free Diet

Life-long adherence to a strictly gluten-free diet (GFD) is currently the only effective treatment for celiac disease (CD), which is an autoimmune enteropathy caused by permanent intolerance to gluten proteins in genetically susceptible individuals [138]. Gluten is a complex mixture of different ethanol-soluble proteins, mainly rich in glutamine and proline residues (prolamins and glutelins) found in grains, such as wheat, rye, oat, and barley [139]. The prevalence of CD is increasing and it is estimated to be about 1% in the European and North American populations, where gluten consumption is estimated to be 10–20 g per person per day [140].

Several studies [141,142,143] clearly highlighted the association between CD and altered gut microbiota, characterized by a higher number of total bacteria and a lower ratio of beneficial/harmful bacteria. Nadal et al. compared intestinal mucosal-associated microbiota in children with active CD (still not on GFD) vs. a control group of healthy subjects [141]. Gram-negative bacteria were significantly higher in the active celiac disease group. In particular, *Bacteroides* spp. and *E. coli* were more abundant in patients with active CD than in the control individuals. Such bacterial taxa were not different between the control individuals and symptom-free patients that consumed a GFD.

Sanchez et al. [142] reported reduced diversity in the genus *Bacteroides*, yet with an increased count of total *Bacteroides*, both in untreated and treated CD children compared with healthy age-matched controls. Of note, the observed dysbiosis does not correlate with the presence of histologic lesions; hence, nutrient malabsorption cannot be considered as the only cause for the reported microbial changes. Furthermore, duodenal *Bacteroides* spp. profiles of patients with treated CD were more similar to those of patients with active CD than the control children. Thus, GFD does not lead to a complete restoration of the balance of *Bacteroides* species in the duodenal mucosa [142]. This result is in line with what has been observed by Di Cagno et al. in their study [143].

Other studies have demonstrated that the total number of beneficial bacteria (i.e., bifidobacteria, lactobacilli, and *Streptococacceae*) is lower in CD patients than in healthy controls. A pediatrics study evaluated fecal samples of patients with active and non-active CD (30 and 18, respectively) vs. age-matched control individuals (*n* = 30). For a deeper analysis, 25 duodenal biopsies from children with active CD, and 8 from nonactive CD vs. 8 from age-matched control individuals were also compared. It was reported that *Bifidobacterium* spp. were lower in patients with active and non-active celiac disease both in the gut lumen and duodenal biopsies. [144].

As far as the effects of GFD on the composition of the gut microbiota, they depend on whether the population undertaking a GFD is healthy or has CD. Indeed, in healthy subjects, GFD negatively affects the gut microbiota. It is associated with a global reduction in the relative abundance of the genera *Bifidobacterium* (particularly *Bifidobacterium longum*) and *Lactobacillus*, and with an increase in the family *Enterobacteriaceae* (particularly *Escherichia coli*-encompassing opportunistic species [145]. In the CD population, the results are quite scarce and controversial. Nadal et al. demonstrated that a GFD is able to restore microbiota in treated CD children, resulting in no significantly different composition compared to the control [141]. On the contrary, Sanchez et al. observed that *Bacteroides*-related dysbiosis persists both in treated CD and untreated CD children [142]. Concerning adult CD patients, Golfetto et al. [146] evaluated the relative abundance of fecal bifidobacteria in 42 healthy subjects vs. 14 celiac patients in GFD for at least two years. The results showed that treated CD patients display depleted bifidobacteria compared with healthy subjects. These findings suggested that an imbalance in the intestinal microbiota of CD patients, due to a reduction in the bifidobacteria, may be a co-factor in triggering the disease [146].

Thus, in CD, the depletion of probiotic species (i.e., lactobacilli and bifidobacteria) and the relative increase in pro-inflammatory bacteria (i.e., Bacteroidetes and Proteobacteria) represent common microbiota fingerprints. However, it is not fully clear whether the dysbiosis is involved in CD pathogenesis by modifying the host immunity and physiology (the “leaky gut dysbiosis” hypothesis) or vice versa, whether CD promotes an altered gut microbiota that can increase inflammation and promote disease progression. It is evident that a complex interplay between genetics, gluten consumption, and environmental factors exists, and more studies are required to elucidate the causative relationships [147,148].

In conclusion, it is evident that GFD reduces bacterial richness [147], with different outcomes depending on the target population. Indeed, in the CD population, it acts by improving gastrointestinal symptoms, restoring a healthy microbial population and reducing pro-inflammatory species; on the contrary, if a GFD is adopted by healthy subjects, a depletion of beneficial species in favur of opportunistic pathogens has been reported [147].

Further studies, particularly in the pediatric population, are needed in order to improve the current knowledge on the effects of GFD on the gut microbiota and to identify the possible biomarker bacteria involved in CD.

### 6.3. Low-FODMAP Diet

The low-FODMAP diet (L-FD) is characterized by the exclusion of certain foods containing fermentable oligo-, di-, mono-saccharides, and polyols. It was proposed by Monash University as a possible therapeutic alternative for several GI disorders. When a L-FD is indicated as a potential treatment, patients are usually advised to exclude FODMAPs for 6 to 8 weeks and then to re-introduce one carbohydrate group at a time. The L-FD consists of the following three phases: exclusion, re-introduction and maintenance, as the exclusion phase is not to be maintained indefinitely. In the adult population, however, a two-fold increase in the ratio of Firmicutes/Bacteroidetes was found in individuals following a L-FD [149]. This finding has been confirmed by other studies, in which the abundance of Firmicutes was enriched, together with a reduced abundance of Bacteroidetes in subjects with IBS compared with healthy subjects [150,151]. However, these effects were only short term. Instead, there was a marked reduction in the relative abundance of *Bacteroides* spp. and *Fusobacterium* in patients that responded to a L-FD and a reduction in bacteria typical of carbohydrate fermentation was found [152]. These same authors suggest quantifying SCFAs prior to a FODMAP to precisely identify the patients who will respond to the intervention, having noted that those who responded the best to the diet were those who had a different fermentability index and high colonic methane and SCFA production [153,154].

This dietary intervention in children is not yet mandatory when IBD occurs. From a recent ESPGHAN position paper [155], only seven randomized control trials have evaluated this diet in children. One study included an assessment of the composition of the gut microbiota and another one evaluated the possible responders to L-FD [156,157]. Twelve children with pediatric Rome III-defined IBS were enrolled in a pilot study [156]. Children followed a one-week L-FD. At baseline, the subjects’ stools were composed mainly of taxa belonging to the *Bacteroidaceae*, *Ruminococcaceae*, and *Lachnospiraceae* families and related orders. The distribution of these taxa did not change significantly after exposure to the LF-D for all subjects; however, overall trends toward increased abundances of members of Clostridiales and decreased abundance of Bacteroidetes were observed following the LF-D intervention [156].

In the follow-up research, the same authors enrolled 33 children with IBS who followed LF-D versus a typical American childhood diet for a week [157]. The results showed that both the baseline gut microbiota composition and the microbial metabolic capacity were associated with the LF-D efficacy. Indeed, the analysis showed that the responder subjects had a higher saccharolytic metabolic capacity within the *Bacteroidaceae* family (e.g., *Bacteroides*), Clostridiales order (e.g., *Ruminococcaceae*, *Dorea* spp., and *Faecalibacterium prausnitzii*), and *Erysipilotrichaceae*. Non-responders were uniquely enriched at the baseline with the genus *Turicibacter*. These findings suggest that the identification of microbiota with greater saccharolytic capacity may serve as a biomarker of responsiveness to a low-FODMAP diet in children, when it is possible to consider this treatment. Thus, further randomized controlled trials, with a bigger sample size and in targeted pediatric populations, are required to elucidate the real impact of LF-D on gut microbiota.

### 6.4. Inborn Errors of Metabolism (IEMs) Diets

Inborn errors of metabolism (IEMs) account for a significant percentage of illnesses in children [158] and represent a complex and dynamic system model in which an entire network of metabolic fluxes operating in a living organism could be altered. This occurs mainly upon a single gene mutation, but it may also be caused by different factors, among which host gut microbiota plays a key role in the disease phenotype [159,160,161].

The management of these diseases usually consists of an exclusion dietary regimen that can reduce gut microbial biodiversity by promoting a state of dysbiosis [162,163]. Hence, IEMs represent a good model to evaluate how special diets are related to specific bacteria profiles that, in turn, could be associated with a characteristic clinical phenotype (“enterophenotype”) [164].

Only a few studies in the literature have reported on the association between gut composition and IEMs, focusing particularly on phenylketonuria (PKU), and studies focusing on the pediatric population are scarce.

PKU is an autosomal recessive disorder in which the impairment of the activity of the enzyme phenylalanine hydroxylase leads to the accumulation of Phe in the blood, which becomes toxic to the brain [165]. To keep Phe concentrations within safe limits and to avoid neurological damage [166], a special “low Phe” diet (based on natural protein restriction, low-protein foods (LPs), and Phe-free L-amino acid supplements) should be started no later than a few days after the positive newborn screening [167,168,169,170].

A Brazilian case–control study was the first to compare the gut microbiota composition of PKU children, with an average age of 4 years, with that of healthy controls using metagenomic techniques [171]. PKU patients more often eat proteins of low biological value (plant rather than animal sources), high rates of low protein food (LPF), and low amount of lipids (monounsaturated and polyunsaturated fat). This resulted in a reduced abundance of the phyla Firmicutes of families *Clostridiaceae*, *Erysipelotrichaceae*, and *Lachnospiraceae*, and of the genera *Clostridiales*, *Coprococcus*, *Dorea*, *Lachnospira*, *Odoribacter*, *Ruminococcus* e *Veillonella* and, in contrast, an increase in *Prevotella* spp., *Akkermansia* spp., and *Peptostreptococcaceae* compared to the control group [171].

Moreover, the PKU microbiome had fewer genes, and thus fewer bacterial functions involved in starch and sucrose metabolism, glycolysis-gluconeogesis, and amino acid biosynthesis. Because different bacteria possess different metabolic pathways to break down sugars [172], lower intestinal production of SCFAs, which provides positive anti-inflammatory effects, was observed [173].

Confirming this data, Verduci et al. reported that, compared with mild iperphenilalaninemia (MPH) subjects, PKU children of a median age of 8.7 years old had lower microbial diversity and a decreased total content of SCFAs, and in particular, butyrate, due to a reduction in some beneficial species, namely *Faecalibacterium* spp. and *Roseburia* spp., which are their main butyrate-producers [169]. In a follow-up study, the same research group confirmed that the greatest differences in the microbial composition between PKU and MHP children were within the Firmicutes phylum. In fact, PKU subjects showed depleted *Faecalibacterium*, *Ruminococcaceae*, and *Veillonellaceae* levels, and were enriched in the genera *Blautia* and *Clostridium* (family *Lachnospiraceae*) and *Lachnospiraceae* (other). Thus, changes in dietary pattern affect the variety of substrates used for microbial fermentations. This results in a reduction or depletion of the specific genera that populate the gut microbiota [174].

However, alterations in the microbiota related to the use of low-protein products and protein substitutes have also been demonstrated. Indeed, starches high in amylose and soluble fibers and free amino acid (AA) formulas could increase SCFA production [175]. Among AAs metabolites, butyrate and indole were shown to have a positive impact on the physiology of the intestinal epithelium, whereas ammonia has a negative impact [176,177]. In addition, it has been observed that glutamate increases microbial diversity and promotes intestinal colonization by *Faecalibacterium* spp. and *Roseburia* spp. [178]. To date, glycomacropeptide (GMP), a natural protein used as a Phe-free-aa supplement [179], seems to provide anti-inflammatory effects by reducing *Desulfovibrio* bacteria and increasing SCFA production in rodents [180] and to have a positive effect on macrobiotic composition by increasing beneficial bacteria, such as *Agathobacter* and *Subdoligranulum,* in children [181].

Concerning the other congenital metabolic diseases, it should be noted that there are only a few studies in the literature on adult cohorts. Data report that drug treatment, in the case of tyrosinemia and alkaptonuria, may have an influence on the composition of the gut microbiota [182,183,184,185,186,187,188], rather than diet and vitamin supplementation with betaine and vitamin B in homocystinuria, leading to an increase in the *Eubacterium coprostanoligenes* group and a decrease in the genera *Alistipes* and *Parabacteroides* [183]. In addition, few studies on adults with GSD Ia and Ib have evaluated how dietary treatment, based on small frequent meals with a high intake of complex carbohydrates (partly provided by uncooked cornstarch) needed to prevent fasting hypoglycaemia, hepatomegaly, and secondary metabolic complications, could influence the availability of substrates for microbial fermentation [185]. GSD subjects presented a significant reduction in gut microbiota biodiversity and were characterized by an increase in the relative abundance of *Enterobacteriaceae* and *Veillonellaceae*, and a reduction in the beneficial genera *Faecalibacterium* and *Oscillospira* spp. Quantification of SCFAs revealed a reduction in SCFA-producing bacteria [187] and a significant increase in fecal acetate and propionate in GSD subjects. These results were paired with a reduced beneficial role, probably due to the unbalanced bacterial interactions [186].

Further studies on the relationship between IEMs and gut microbiota are needed, not only to understand the pathophysiology of these diseases and the development of new biomarkers and therapies but also to improve the long-term quality of life of affected patients [162].

### 6.5. Ketogenic Diets

The ketogenic diet (KD) is a high-fat diet with adequate protein intake and very low carbohydrate content. It can mimic the metabolic effects of the fasting state, without significant caloric deprivation, to induce the production of ketone bodies [189].

KD has been considered a successful dietary approach for the treatment of epilepsy since 500 B.C.; however, the past 15 years have observed a tremendous increase in KD use and in scientific interest, especially for children with difficult-to-control epilepsy [190]. Since 1998, the effectiveness of the KD, both in terms of reducing the frequency and improving seizure control, has been demonstrated in both observational and retrospective studies and randomized controlled trials conducted in children with drug-resistant epilepsy myoclonic epilepsy (Dravet’s syndrome) [191] and myoclonic-atonic epilepsy (Doose’s syndrome) [192]. Recent studies in mouse seizure models revealed that the gut microbiome associated with epilepsy was altered, but it could change in its composition upon KD administration [193]. Modifications in the gut microbiota of children with drug-resistant epilepsy after a ketogenic diet have been investigated in many studies, but bacterial changes vary from report to report. Xie and colleagues [194] demonstrated a decrease in the microbiota diversity as early as after 1 week of KD. They observed a reduction in Proteobacteria, a persistence of low numbers of *Cronobacter*, and an increase in *Bacteroides*, especially in *Prevotella*, and in *Bifidobacterium* spp. The authors concluded that the microbiota of infants with epilepsy differed from that of healthy controls, with an increase in beneficial bacteria and a decrease in pathogenic ones [194]. The efficacy of the KD in seizure control might depend on the ability of specific bacterical populations to alter gamma glutamylation of amino acids, and thus modify their uptake at the central nervus system level [195].

Tagliabue et al. did not report changes in Bacteroidetes and Firmicutes but highlighted a significant increase in *Desulfovibrio* spp. upon 3 months of KD [196]. However, an interesting exploratory pilot study on the microbial profile of drug-refractory children with epilepsy reported a reduced diversity with a depletion in Firmicutes and Actinobacteria and enrichment of Bacteroidetes following 6 months of KD, compared with untreated patients. The effect of diet was also different among patients. Indeed, participants in this study showed different responses to seizure reduction, and those who did not respond to treatment had persistently elevated levels of *Alistipes*, *Clostridiales*, *Lachnospiraceae*, *Ruminococcaceae*, and *Rikenellaceae,* compared with those who positively responded to treatment [197].

In addition, Lindefeldt and colleagues studied the taxonomic and functional profiles of the gut microbiota of 12 children with drug-resistant epilepsy, using a shotgun metagenomic approach, at baseline and after 3 months of KD. After treatment, the authors found a decrease in the relative abundance of *Dialister* spp., *Bifidobacterium* spp., and *Eubacterium rectale* and a parallel increase in the relative abundance of *Escherichia coli*, compared with the control, leading to an overall reduction in the relative number of beneficial fiber-consuming bacteria [198].

This evidence suggested that the microbiome differs significantly in patients upon KD with regard to the diversity and abundance of specific bacterial species, which in turn may be considered as possible therapeutic targets and biomarkers for the effectiveness of therapy in children with refractory epilepsy [197,198].

Considering the low number of studies that address this topic and the small sample size of the enrolled cohorts, comprehensive multicentre studies are needed to clarify the KD efficacy and whether its therapeutic effect is sustained and/or can be maintained after diet discontinuation by dietary supplements, such as prebiotics and probiotics [199]. Lastly, the very low carbohydrate ketogenic diet (VLCKD) adopted during pregnancy and lactation seems to have a protective effect on the development of infants’ gut microbiota [200] and in early childhood, a in reduction in asthma insurgence [201]. This relies on the high intake of PUFA, vegetable protein, dietary fibers, linoleic (ω-6) fatty acid and polyphenols that stimulate the relative abundance of SCFA-producing bacteria. However, it is crucial to assess the safety of this regimen during pregnancy and lactation, and to fully elucidate its influence on the forming gut microbiota. Further randomized control trials would also be needed to elucidate the mechanisms by which SCFA-producing bacteria exert their anti-inflammatory effects in adipose tissue in early childhood.

## 7. Conclusions

The gut microbiota plays a fundamental role in the health and well-being of a growing child. A combination of habits, such as lifestyles and dietary patterns, may influence its richness and diversity in early life. Overall, a varied and healthy diet appears to confer a positive modulation of gut microbiota. However, there are pathological conditions for which it is necessary to adopt a dietary regimen from early childhood. These patterns, with specific macronutrient ratios or without certain nutrients (or compounds), may alter the gut microbiota. Often, these bacterial shifts, compared to a healthy child’s microbiota, could trigger the overgrowth of bacteria-linked intestinal inflammation [100]. Diets that decrease SCFA-producing bacteria, such as the GFD, PKU diet, and KD and those with low-level consumption of a plant-based food diet, may have negative effects on the host. Similarly, dietary patterns that increase the presence of Proteobacteria (Western diet or GSD diet) may lead to increased systemic inflammation, also due to elevated cytokine production [202].

From our point of view, this is the first review to report the impact of several dietary patterns on the gut microbiota of infants and children under healthy or pathological conditions.

Alteration in microbial taxa that colonize the gut during infancy and childhood could result in impaired host–microbe interactions throughout life, leading to deleterious consequences, such as a greater predisposition to non-communicable diseases, an unbalanced immune system, an altered microbiota-gut-brain communication, and possible future cognitive decline. When the exclusion of certain foods from the diet is mandatory for pathological reasons, the greatest challenge of identifying specific biomarkers (such as metabolites) for microbial alterations remains. Such biomarkers may become a valuable tool for verifying the adherence to the diet, its efficacy and also the early identification or prevention of intestinal disorders. Therefore, as a first step, upcoming studies on pediatric populations affected by the diseases discussed above are crucial to define specific enterotypes and detect their alterations over time.

In the pediatric age, elimination diets are often adopted even without the presence of overt pathology. Embracing a special diet without medical consultation by a specialized team may not be beneficial for the child. Thus, research should be focused on the possibility of identifying the so-called “responders” to a certain dietetic therapy in order to suggest personalized nutrition that also encompasses gut microbiota health.
